# Cellular, Molecular and Proteomic Characteristics of Early Hepatocellular Carcinoma

**DOI:** 10.3390/cimb44100322

**Published:** 2022-10-10

**Authors:** Athanasios Armakolas, Vasiliki Dimopoulou, Adrianos Nezos, George Stamatakis, Martina Samiotaki, George Panayotou, Maria Tampaki, Martha Stathaki, Spyridon Dourakis, John Koskinas

**Affiliations:** 1 Physiology Laboratory, Athens Medical School, National and Kapodistrian University of Athens, 115 27 Athens, Greece; 2 B’ Department of Medicine, Hippokration Hospital, National and Kapodistrian University of Athens, 115 27 Athens, Greece; 3 Institute for Bioinnovation, Biomedical Sciences Research Center “Alexander Fleming”, 166 72 Vari, Greece

**Keywords:** HCC, CTCs, miRNAs, proteomics

## Abstract

Hepatocellular carcinoma (HCC) accounts for the majority of primary liver cancers. Early detection/diagnosis is vital for the prognosis of HCC, whereas diagnosis at late stages is associated with very low survival rate. Early diagnosis is based on 6-month surveillance of the patient and the use of at least two imaging modalities. The aim of this study was to investigate diagnostic markers for the detection of early HCC based on proteome analysis, microRNAs (miRNAs) and circulating tumor cells (CTCs) in the blood of patients with cirrhosis or early or advanced HCC. We studied 89 patients with HCC, of whom 33 had early HCC and 28 were cirrhotic. CTCs were detected by real-time quantitative reverse transcription PCR and immunofluorescence using the markers epithelial cell adhesion molecule (EPCAM), vimentin, alpha fetoprotein (aFP) and surface major vault protein (sMVP). Expression of the five most common HCC-involved miRNAs (miR-122, miR-200a, miR-200b, miR-221, miR-222) was examined in serum using quantitative real time PCR (qRT-PCR). Finally, patient serum was analyzed via whole proteome analysis (LC/MS). Of 53 patients with advanced HCC, 27 (51%) had detectable CTCs. Among these, 10/27 (37%) presented evidence of mesenchymal or intermediate stage cells (vimentin and/or sMVP positive). Moreover, 5/17 (29%) patients with early HCC and 2/28 (7%) cirrhotic patients had detectable CTCs. Patients with early or advanced HCC exhibited a significant increase in miR-200b when compared to cirrhotic patients. Our proteome analysis indicated that early HCC patients present a significant upregulation of APOA2, APOC3 proteins when compared to cirrhotic patients. When taken in combination, this covers the 100% of the patients with early HCC. miR-200b, APOA2 and APOC3 proteins are sensitive markers and can be potentially useful in combination for the early diagnosis of HCC.

## 1. Introduction

Liver cancer is the second-most lethal form of cancer, with a 5-year survival rate of 18% [1]. It is the sixth-most common cancer in terms of incidence rate, with nearly 800,000 cases reported annually. Recent evidence suggests that liver cancer incidence is increasing, with a mortality rate estimate of more than 1 million in 2030 [1,2]. Hepatocellular carcinoma (HCC) accounts for the majority of primary liver cancers. Early detection and diagnosis are vital for the prognosis of the disease, whereas in patients with HCC that are diagnosed at late stages, the odds are dismayingly low. Survival rates for advanced HCC vary by country, with the one-year survival rate being approximately 20% after diagnosis [1,2,3]. The majority of HCCs occur in patients with underlying liver disease, usually as a result of chronic hepatitis B or C virus (HBV or HCV) infection, alcohol abuse or metabolic syndrome [4].

Chronic hepatitis B virus (HBV) and hepatitis C virus (HCV) infections are major global public health problems, with an estimated 1 million and 450,000 deaths yearly worldwide, respectively [5]. Up to 40% of patients with chronic hepatitis B virus infection and approximately 20–30% of subjects infected with HCV are estimated to develop liver cirrhosis. Most commonly, deaths occur from complications of liver cirrhosis, namely liver failure and HCC [5].

In the absence of antiviral therapy, the prognosis for patients with HBV cirrhosis is poor, with a 5-year survival of only 14%, compared to 84% in patients with compensated HBV cirrhosis. Similarly, patients with HCV cirrhosis in the absence of anti-viral therapy have a 67%–91% mortality rate due to liver-related causes, including HCC or hepatic failure [6]. However, patients with HCV cirrhosis, even if cured of HCV infection, maintain a significant risk of HCC development [6,7].

Non-alcoholic steatohepatitis (NASH) as a liver manifestation of metabolic syndrome and obesity is rapidly increasing and is expected to become the predominant risk factor for HCC in high-income regions in the coming years [7].

It is estimated that 2–5% of patients with cirrhosis will develop HCC annually [1,2]. For early detection of HCC, it is recommended that all patients with cirrhosis should be under 6-month surveillance with ultrasound by an experienced radiologist. In the presence of a nodule, a contrast-enhanced CT or MRI should be performed. A distinctive pattern of hyper-enhancement in the arterial phase and washout in venous or delayed phases is diagnostic for HCC. MRI screening for small HCC (≤3 cm) has 78.82% sensitivity, 78.46% specificity, 78.67% accuracy, 82.72% positive predictive value and 73.91% negative predictive value [8]. For patients with nodules that are less than 1 cm in diameter, follow-up imaging is mandatory [2,9].

Since early diagnosis is vital for the prognosis of HCC, it is easy to understand the necessity of the development of diagnostic tools for the early diagnosis of HCC in cirrhotic patients. In this study, we investigated various biological markers in the blood of patients with early and advanced HCC and cirrhosis without HCC. The markers examined were: circulating tumor cells (CTCs) of epithelial (epithelial cell adhesion molecule, (EPCAM), mesenchymal (vimentin) and intermediate stage (surface major vault protein) subtypes and a set of microRNAs (miRNAs) that are involved in HCC [10,11,12,13]: miR-122, which is associated with metastasis and poor prognosis [2]; miR-200a and miR-200b, which have been found as early markers of HCC development [14,15]; and miR-221 and miR-222, which have been shown to target the cell-cycle-dependent kinase inhibitor p27 and induce cell proliferation, metastasis and infiltration [15,16]. The blood of all these patients was also examined for protein signatures using whole proteome analysis.

Previous evidence indicated the possibility of the use of multi-omics techniques prior to the establisment of more accurate diagnostic tools in HCC [17,18]. Our aim was to determine useful markers to predict the development of HCC at very early stages in cirrhotic patients.

## 2. Materials and Methods

### 2.1. Patient Selection

A total of 117 patients were included in the study—mean age 65 ± 11 years, 87 (74%) males. With respect to the etiology of the underlying liver disease, 41 (35%) cases were viral-related, 39 (33%) alcohol- and/or metabolic-syndrome-related and 37 (31.6%) related to other etiology (Table 1).

Concerning the presence of HCC, 89 patients had non-treated HCC (56 patients with advanced and 33 with early HCC) and 28 were age-matched cirrhotic patients with no evidence of HCC, based on imaging studies and normal serum aFP levels. Five healthy controls were also recruited in the study. Total cellular RNA, serum miRNA and serum proteins were extracted from the five healthy individuals, and the average value obtained in each case was used as the reference value. 

Early HCC was defined as a nodule ≤3 cm. Diagnosis of HCC was based on MRI-characteristic hemodynamic pattern and/or liver biopsy. Whole blood, plasma and serum samples were obtained from all patients.

The study was approved by the National and Kapodistrian University of Athens Medical School Ethics Committee (Approval code: 029/19.11.18). All subjects gave informed consent in accordance with the Declaration of Helsinki. Written informed consent was obtained from every patient and healthy individual involved in this study. All methods were performed in accordance with the relevant guidelines and regulations. The relatively small number of patients included in the study is mainly due to the prospective nature of the study, the availability of patients in the out-patient clinic and the restricted sample collection time frame in order to obtain a meaningful follow-up period.

### 2.2. CTCs

Cells were isolated from 10 mL peripheral blood, and after treatment with erythrocytes lysis buffer, the pellet was treated with TRIZOL reagent according to the manufacturer’s instructions. Briefly, 2.5 mL freshly isolated peripheral whole blood samples was mixed with 7.5 mL erythrocyte lysis buffer (ELB) and incubated on ice for 30 min, followed by centrifugation 400 g/10 min/4 °C and lysis of the cell pellet using TRIzol Reagent (Cat # 15596026, Thermo Scientific, Waltham, MA, USA). RNA extraction was performed according to the manufacturer’s instructions and stored at −80 °C. The quantity and quality of RNA samples was spectrophotometrically recorded (Biospec Nano, Kyoto, Japan).

### 2.3. MicroRNAs

Plasma miRNA was extracted with a Nucleospin miRNA plasma kit (Cat # 740971, MACHEREY-NAGEL GmbH & Co. KG, Duren, Germany) according to manufacturer instructions. 

### 2.4. cDNA Synthesis and Real-Time PCR

A Mir-X miRNA First-Strand Synthesis kit (Cat # 638316, Takara Bio Inc., Otsu, Shiga, Japan) was used for the synthesis of first-strand cDNA from our purified miRNA samples according to the manufacturer’s instructions. Complementary DNA samples were diluted 1:5 with nuclease-free water (Qiagen, Hilden, Germany) immediately after synthesis and stored at −20 °C.

Total RNA (0.5μg) obtained from peripheral whole blood samples was reverse transcribed using PrimeScript RT Reagent Kit (Perfect Real Time) (Cat # RR037A, Takara Bio Inc., Otsu, Shiga, Japan). Complementary DNA samples were diluted 1:10 with nuclease-free water (Qiagen, Germany) immediately after synthesis and stored at −20 °C.

Quantitative real-time polymerase chain reaction (qRT-PCR) was carried out using the Bio-Rad IQ5 thermocycler and the Kapa Biosystems SYBR Green (Cat # KR0389, Kapa Biosystems, Cape Town, South Africa).

Specific 5΄ primer to amplify only miR-122, miR-221, miR-222, miR-200a and miR-200b was synthesized according to Mir-X miRNA First-Strand Synthesis kit instructions and the miRBase Sequence Database (University of Manchester: http://www.mirbase.org assessed on 20 May 2018). Primer sequences are shown in Supplementary Table S1. As a normalization miRNA, U6 snRNA control (reference) was used, supplied by the Mir-X miRNA First-Strand Synthesis kit. Despite the fact that the quantitative determinations of the miRNAs examined were not obtained using probes, the specificity of our assay was induced by the isolation of only small-size RNAs from plasma; the primers were selected under stringent conditions, and they were specific for only one target; and the PCR conditions were set prior to favor the amplification of small molecules.

Specific primers to amplify only cDNA (exon-intron spanning) for circulating-tumor-cell-related genes: MVP, EPCAM, vimentin, aFP and the normalization (reference) gene GAPDH were designed using the Beacon Designer software. The sequences of each primer set are shown in Supplementary Table S1.

The reaction was performed in a total volume of 20 μL per reaction and constituted of 2 μL of template cDNA, 0.4 μM of each primer, 10 μL 2× KAPA SYBR Green Mix (Kapa Biosystems, Cape Town, South Africa) and ultra-pure water. A two-step amplification protocol was applied, starting with step 1, with one cycle at 95 °C for 4 min followed step 2, with 40 cycles at 95 °C for 5 s and 63 °C for 30 s. The specificity of the amplified products was determined via melting curve analysis.

The threshold cycles (Ct) generated by the qPCR system were used to calculate relative gene expression levels between different samples [13,19]. Briefly, the Ct of the target gene was subtracted from the Ct of the reference gene for the two groups, and the relative expression of each sample was determined using the 2^−ΔΔCt^ method. All reactions were performed in duplicate. The average value of the samples from the HC group in each case was used as the reference sample.

The average calibrator ΔCq value in EPCAM was obtained from these samples, and individual ΔCqs of the calibrator were within 2 SDs of the average calibrator ΔCq value. Samples were classified as positive for a particular gene if the 2^−ΔΔCq^ was 2.0 or more (i.e., 100% or more than what is found in healthy blood) [19]. Additionally, prior to the determination of the cutoff points that would define positive from negative patients in the case of CTC and miRNA markers’ ROC, curve analysis was carried out using biomarkers’ continuous variables (Figure S1).

### 2.5. Immunofluorescence Staining (IF)

Peripheral blood (10 mL) was centrifuged, and the cells were resuspended in RPMI 10% FBS. The cells were then cultured on chamber slides for 6 hrs, and they were stained by a direct immunofluorescence method. Cells were rinsed in PBS and fixed with ice-cold 80% methanol for 10 min at room temperature. Firstly, they were stained with anti-EPCAM and anti-MVP fluorescent primary antibodies for 1 hr at room temperature, and they were then permeabilized with 0.5% Triton X-100 (Cat # 9002-93-1, Sigma Aldrich, St. Louis, MO, USA) for 10 min. They were then incubated with the anti-vimentin fluorescent primary antibody again for 1 h at room temperature. The antibodies used were Texas Red conjugated anti-EPCAM (1:100), Alexa fluor 480 anti-Vimentin (1:100) and Alexa fluor 647 anti-MVP (1:100) (Cat # ab286811, ab195877, ab208627, respectively, all from Abcam, Cambridge, UK). After 3 washes, samples were stained with 4′,6-diamidino-2-phenylindole (DAPI) (1 μg/mL) for viewing with a microscope (Olympus BX40, Tokyo, Japan). Patients were considered positive for the existence of CTCs when more than 10 EPCAM-positive cells were detected in 5 mL of blood.

### 2.6. Proteome Analysis

#### Liquid Chromatography Mass Spectrometry (LC MS)

For each patient and normal sample, 30 μL of serum was analyzed. Peptides were separated with an Acclaim Pepmap 75 μm × 50 cm on an Ultimate 3000 nano LC system set at 40 °C. The HPLC was performed using 350 nl/min flow with 0.1% formic acid in water as solvent A and as 0.1% formic acid in acetonitrile as solvent B. For each injection, 500 ng was loaded and eluted with a linear gradient from 8% to 24% buffer B over 50 min; then, the gradient was ramped up to 36% buffer B over 10 min. The column was washed with 100% buffer B for 5 min and was left to equilibrate to 8% buffer B for 15 min.

The gas phase fractionation (GFP) of the pooled sample was performed using 12 × 50 m/z windows ranging from 400–1000 m/z at 60,000 MS1 with an AGC of 3 e6 for 60 ms and MS2 at 30,000 with an AGC of 1 × 10^6^ for 60 ms; NCE was set to 27, and +2H was assumed as the default charge state. The GPF-DIA acquisitions used 4 m/z precursor isolation windows in a staggered window pattern with optimized window placements.

The DIA conditions for the sample analysis were (a) measured at 390–1010 m/z with 60,000 resolution and an AGC target of 3e6, IT of 60 ms for MS1, and (b) 76 × 8 m/z DIA isolation windows were measured at 15,000 resolution in a staggered-window pattern with optimized window placements from 400 to 1000 m/z using an NCE 0f 27 at a +2 default charge state. 

Data processing proteomics:

The raw files from GFP were overlap demultiplexed with 10 ppm accuracy after peak picking in ProteoWizard (version 3.0.18299). Searches were performed using EncyclopeDIA (version 0.9), which was configured to use default settings: 10 ppm precursor, fragment and library tolerances. EncyclopeDIA was allowed to consider both B and Y ions, and trypsin digestion was assumed.

The created library was imported in skyline (version 20.1) by removing repeated peptides. The raw files were imported and reintegrated by an m-prophet model with 1:1 decoys, and the peptides were filtered for dotp >0.7. The total fragment areas for each peptide were exported, and all peptides corresponding to a protein were summed. The statistical analysis of the samples was performed in Perseus version (1.6.10.0). The total fragment area of each protein from the previous step was normalized to the total area of the sample. 

The mass spectrometry proteomics data have been deposited to the ProteomeXchange Consortium via the PRIDE [1] partner repository with the dataset identifier PXD033306.

### 2.7. Statistical Analysis 

Statistical analysis was performed by SPSS V23 (SPSS software; SPSS Inc, Chicago, IL, USA) and ROC curves were performed using the MedCalc^®^ Statistical Software, version 20.113 (MedCalc Software Ltd., Ostend, Belgium; https://www.medcalc.org; accessed on 21 August 2022). Data were expressed as frequencies, mean ± SD, or median with interquartile range (IQR), as appropriate. Quantitative variables were compared using Student’s *t*-test or Mann–Whitney test for normally distributed and non-normally distributed variables, respectively. Qualitative variables were compared with Chi-squared test or Fisher’s exact-test, as appropriate. The relationships between quantitative variables were assessed using the Spearman’s correlation coefficient. All tests were two-sided, and *p* values < 0.05 were considered to be significant. 

## 3. Results

### 3.1. CTCs

**Figure 1:**
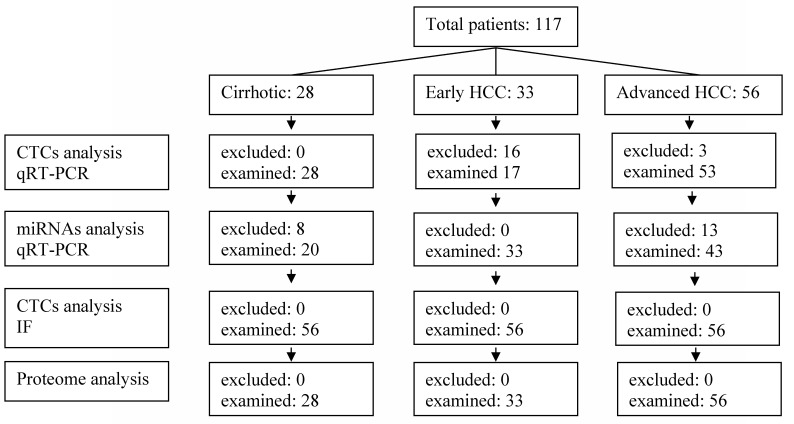
Flow cart of the patients involved in the experimental procedure.

Circulating tumor cells of all types were detected in the serum of patients with HCC of all stages. Detection was obtained by qRT-PCR in total RNA isolated from peripheral blood and by immune-fluorescence in blood cells. Cells and cellular RNA were evaluated in 53 out of 56 patients with advanced HCC, in 17 out of 33 patients with early HCC and in all 28 patients with cirrhosis (Figure 1). Only patients that were positive both for IF and qRT-PCR were considered positive for the existence of CTCs (Figure 1).

**Figure 2:**
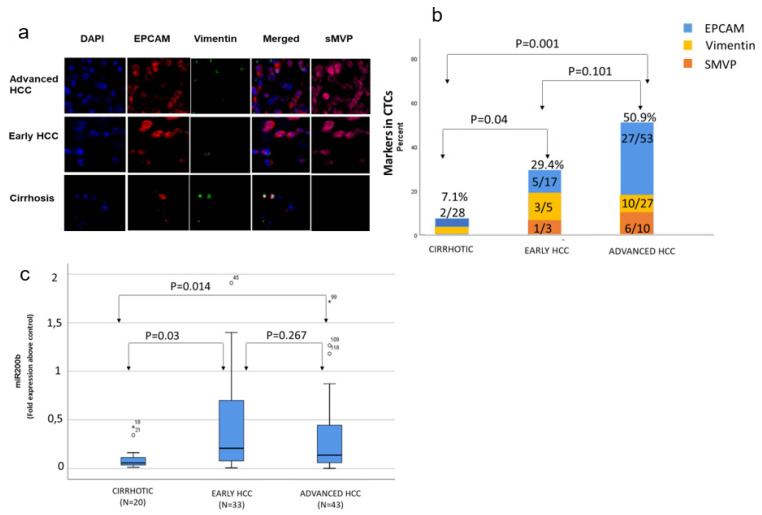
Detection of circulating tumor cells (CTCs) in the blood of cirrhotic and cancer patients: (**a**) detection of CTCs via immunofluorescence in the three different groups of patients.; (**b**) real-time quantitative reverse transcription PCR analysis for the detection of circulating cells expressing epithelial cellular adhesion molecule (EPCAM), vimentin and sMVP (Pearson chi-square test). DAPI: 4′,6-diamidino-2-phenylindole; sMVP: surface major vault protein; HCC: hepatocellular carcinoma. (**c**) Detection of microRNAs (miR) in the blood of hepatocellular carcinoma (HCC) and cirrhotic patients indicated that miR-200b presented significant difference between cirrhotic and early/advanced HCC (Mann–Whitney test), (* *p* < 0.05).

Out of 53 patients with advanced HCC who had been evaluated, 27 (~51%) presented evidence of CTCs (Table 2). Ten patients out of twenty-seven, exhibited mesenchymal-type CTCs, presenting high vimentin and low EPCAM expression. Six of the patients with CTCs of mesenchymal subtype also presented evidence of sMVP too, as was observed by IF, whereas four patients showed sMVP expression (intermediate differentiation) without expressing vimentin. The remaining patients presented CTCs of epithelial subtype expressing only EPCAM (Figure 2a,b).

In patients with early HCC, 5 (29%) out of 17 patients who had been evaluated presented evidence of CTCs. Three out of five showed high vimentin expression, and one out of three additionally presented sMVP expression. Comparison of the actual numbers of CTCs of any type suggested that individuals with advanced cancer presented significantly higher number of CTCs compared to individuals with cirrhosis (*p* = 0.009) (Figure 1a,b). In patients with cirrhosis, 2 (7%) out of 28 presented evidence of CTCs, and 1 of them also presented high vimentin levels (Table 2) (Figure 2a,b). During a 2-year follow-up period, none of these patients developed HCC. 

aFP was measured in the serum and in the blood isolated mRNA of patients. aFP expression in CTCs was detected in 12 out of the 27 EPCAM-positive patients with advanced HCC and in the majority of the cases was associated with the existence of vimentin. Expression of aFP was observed only in one out of five early HCC patients with detectable CTCs who also had elevated serum aFP levels (56 ng/mL). Finally, in cirrhotic patients, aFP expression (qRT-PCR) was observed in one out of two patients with CTCs, and one of these two patients exhibited vimentin expression. Regarding serum aFP levels, only 7 out of 33 (21%) patients with early HCC had elevated levels (>20 ng/mL), in contrast to 42 out of 56 (75%) patients with advanced HCC. None of the cirrhotic patients had elevated serum aFP (Table 1).

The presence and subtypes of CTCs in all groups studied did not correlate to the type of underlying liver disease (viral/non-viral).

### 3.2. MiRNAs 

MiRNAs from the plasma of patients were evaluated in 43 out of 56 patients with advanced HCC, in all 33 patients with early HCC and in 20 out of 28 cirrhotic patients.

Quantitative analysis of the five most important miRNAs associated to diagnosis and prognosis of HCC (miR-122, miR-200a, miR-200b, miR-221 and miR-222) suggested that the only marker that was significantly associated with early HCC was miR-200b. Patients with either early or advanced HCC presented a significant upregulation in miR-200b levels compared to the cirrhotic patients (Figure 1c). Furthermore, miR-200b upregulation was observed in all patients that presented CTCs, and it was proportional to EPCAM elevation as was monitored by qRT-PCR (Table 3). miR-200b level did not correlate to the type of underlying liver disease (viral/non-viral). 

### 3.3. Proteomics

Proteomics were evaluated in all patients of this study; 56 with advanced HCC, 33 with early HCC and 28 with cirrhosis.

**Figure 3:**
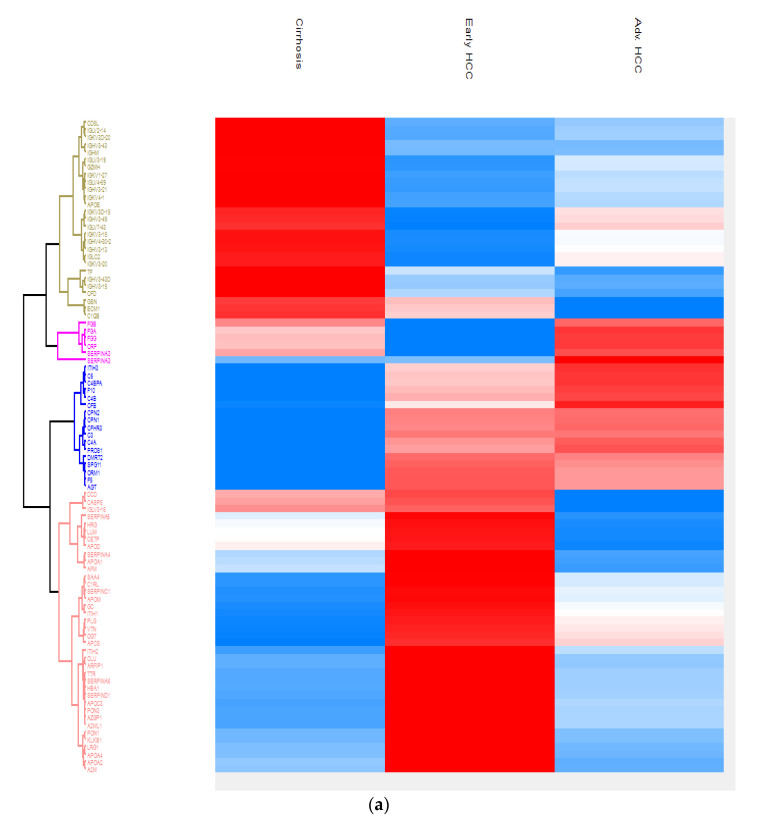

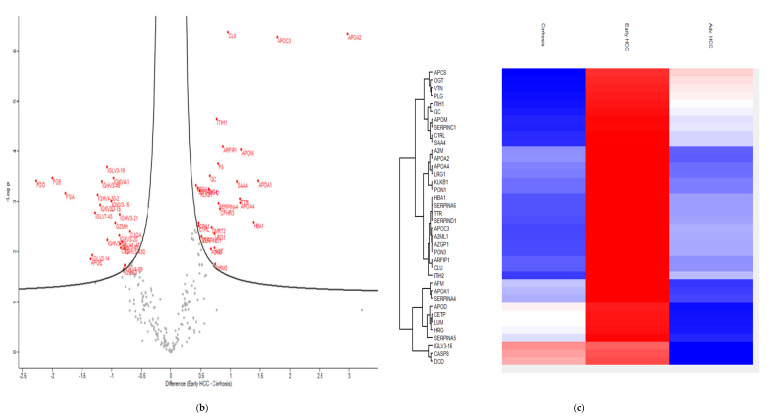
Proteomics comparison of early hepatocellular carcinoma (HCC) vs cirrhotic patients: (**a**) heatmap of the proteomes in all three patient categories; (**b**) volcano plot produced after the comparison of patients with early HCC and cirrhotic patients. At the right side of the plot, the proteins that were upregulated in early HCC patients are observed. The farther away the protein from the lines of the plot, the greater the significance; (**c**) comparison of the 3 patient categories for the 31 proteins determined by the volcano plot.

By defining high-stringency criteria, a total of 228 proteins that show a statistically significant difference in expression between patients with early HCC, cirrhotic patients and patients with advanced HCC were identified (Figure 3a). To determine markers that will be useful for determining early HCC, a proteomic comparison was performed between patients with cirrhosis and patients with early HCC with Student’s *t*-test.

The results were displayed on a volcano plot. A total of 53 proteins were found that can be used as potential biomarkers to determine early HCC from cirrhosis. These proteins were further tested using the Metascape online platform enrichment tool to determine their function, the biological processes involved, their involvement in carcinogenesis and possible protein interactions. Out of a total of 53 proteins, 31 were upregulated and 22 were downregulated in patients with early HCC compared to cirrhotic patients (Figure 3b). The proteins that were up regulated in early HCC compared to cirrhotic were: AGT, APCS, APOA1, APOA2, APOA4, APOC3, APOM, ARFIP1, C1RL, CFHR3, CLU, CPN1, CPN2, DMRT2, F5, GC, HBA1, ITIH1, ITIH2, KLKB1, LRG1, OGT, PON3, PROS1, SAA4, SERPINA4, SERPINC1, SERPIND1, SPG11, TTR and VTN. The significance of these proteins is also presented in a heatmap in comparison to cirrhotic and HCC patients (early and advanced) (Figure 3c).

**Figure 4:**
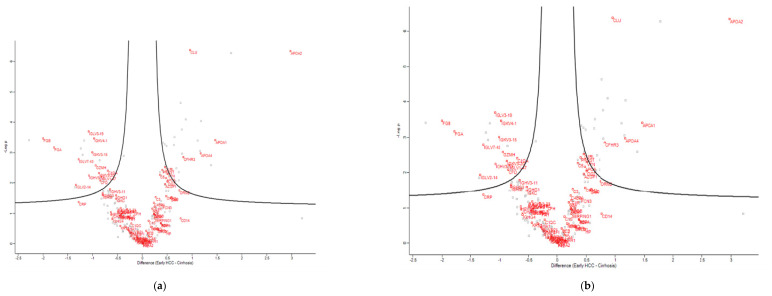

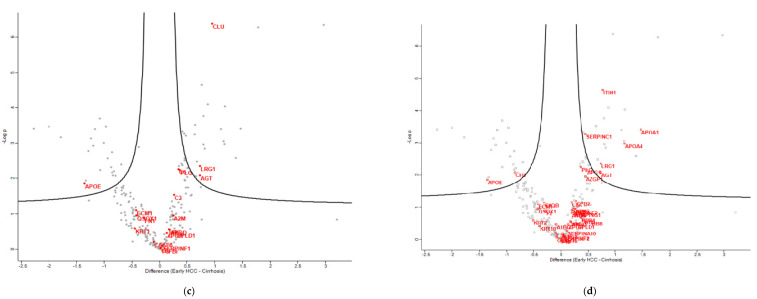

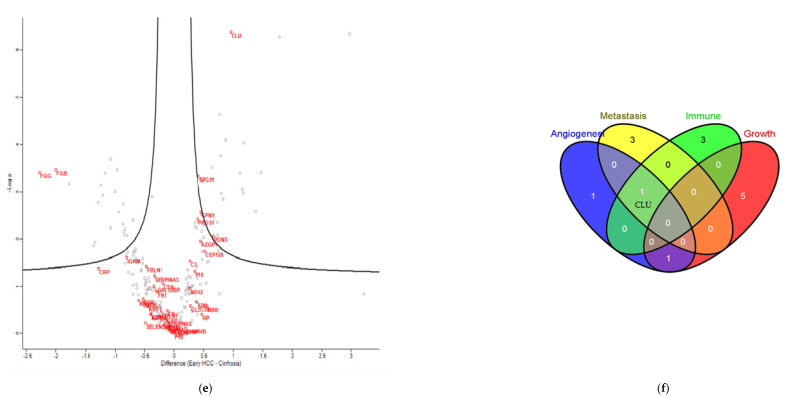
Volcano analyses of cirrhotic vs early HCC patients. The patients are compared for the expression of the proteins involved in: (**a**) growth; (**b**) immune response; (**c**) angiogenesis; (**d**) proliferation; (**e**) metastasis. (**f**) CLU protein (apolipoprotein J) seems to be central in metastases, angiogenesis and immune response.

These proteins were further analyzed in five central mechanisms involved in carcinogenesis: growth (Figure 4a), immune response (Figure 4b), angiogenesis (Figure 4c), proliferation (Figure 4d) and metastases (Figure 4e). Seven proteins were which showed the greatest statistical significance in terms of expression intensity as presented in the thermograms in patients with early HCC as compared to cirrhotic patients were selected. These proteins were APOA2, APOC3, CLU, OGT, APOD, VTN and HRG. CLU protein (apoliporotein J) seems to be a very potent marker for HCC since it presented high statistical significance, and it seems to be involved in many biological processes that are central to HCC (Figure 4b,c,e,f). Examination of each of these markers and the remaining 24 of all the 31 markers that were found to be up regulated in the early HCC patients indicated that none of these markers alone present the sensitivity or the specificity to stand as a diagnostic or prognostic marker. However, the combination of APOA2 and APOC3 upregulation covered 100% of the patients with early HCC. In detail, APOC3 was upregulated in 29/33 (88%) and APOA2 in 28/33 (85%) in early HCC patients. Both markers were also found to be upregulated and in 2/28 cirrhotic patients. 

Protein profile was not affected by the etiology of underlying liver disease. Data are available via ProteomeXchange with identifier PXD033306. 

### 3.4. Clinical Significance of ApoA2, APOC3 and mir-200b

**Figure 5:**
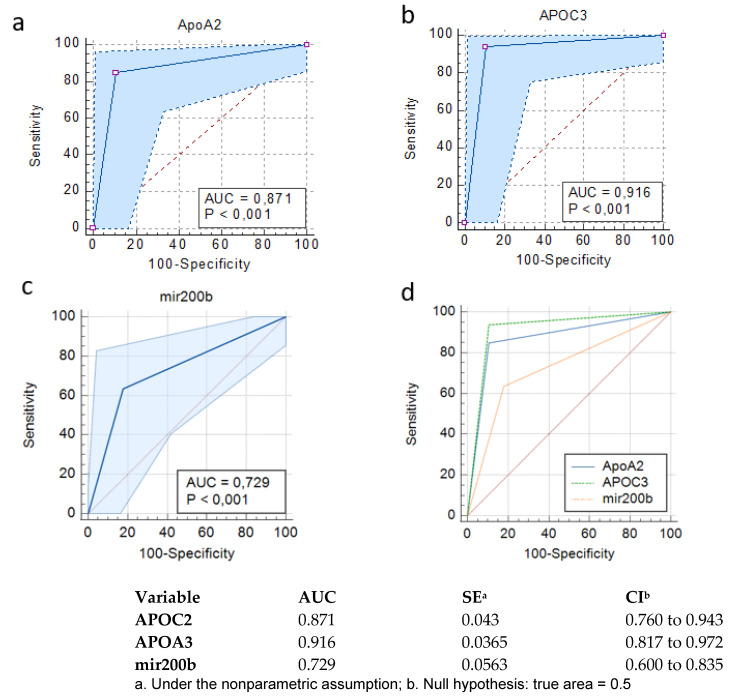
ROC analysis for the determination of specificity and sensitivity of selected markers to define early HCC from cirrhotic patients. (**a**) APOA2, (**b**) APOC3, (**c**) miR-200b, (**d**) the three markers combined.

Receiver operating characteristic (ROC) analysis indicated the significance of both protein markers APOA2 and APOC3. APOA2 presented sensitivity of 84.95% and specificity of 89.29% with an area under the curve (AUC) of 0.81, and APOC3 presented 93.94% sensitivity and 89.29% specificity, AUC= 0.916 (*p* < 0.001 for both markers) (Figure 5a,b,d). miR-200b presented sensitivity of 63.6% and specificity of 82.1%, AUC= 0.729 (*p* < 0.001) (Figure 5c,d). 

## 4. Discussion

Early detection of HCC is associated with better prognosis and a five-year survival of 40–70%, which drops dramatically in intermediate (3-year survival 10–40%) and advanced stages (<12 months) [1,2,3]. Therefore, early detection of HCC is crucial for the survival of these patients. In clinical practice surveillance for HCC, ultrasound (U/S) every 6 months in cirrhotic patients is mandatory for early detection, followed by contrast enhancing images by computed tomography/magnetic resonance imaging (CT/MRI) for confirmation. However, their sensitivity and specificity depend on size and type of HCC. Liver biopsy is an invasive method that is not always feasible and sometimes not diagnostic for various reasons. Having said that, detection of HCC at very early stages is still a challenging task. Recent sophisticated omics techniques have been applied for HCC and have reported possible prognostic models based on molecular signatures, but with low sensitivity and accuracy [17,18,19]. Consequently, the necessity of the development of more accurate and sensitive tools guiding early diagnosis and prognostic assessments in HCC patients remains.

In this study, we combined the power of proteomics, CTCs and HCC-associated miRNAs in the blood of patients with early HCC, advanced HCC and cirrhosis with no obvious HCC, aiming towards the determination of novel and accurate HCC diagnostic markers which could be combined for the development of a novel tool for the diagnosis and/or prognosis of early HCC. 

To our knowledge, the vast majority of studies that aim towards the determination of novel biomarkers in HCC compare cirrhotic patients or healthy controls to HCC patients [17,18,19,20]. In this study, our aim was to investigate possible cellular and molecular differences by analyzing the proteome and miRNA profiles of patients with specifically early HCC in comparison to cirrhotic patients without HCC.

CTC analysis enables early cancer detection, prognosis prediction and therapy response monitoring in patients with HCC. Despite this, translation of CTC analysis from bench to patient is a challenging task mainly due to the fact of the heterogeneity of the studies in respect to detection techniques/technologies and the lack of standardized assays [16]. In this study, we determined epithelial, mesenchymal and intermediate-stage CTCs (EPCAM, vimentin and sMVP, respectively) via qRT PCR and IF. Furthermore, we examined the expression of aFP by qRT-PCR.

Changes in cytoplasmic aFP expression have been found to be associated with the expression of several metastasis-related mesenchymal proteins: keratin 19 (K19), EPCAM, matrix metalloproteinase 2/9 (MMP2/9) and C-X-C motif chemokine receptor 4 (CXCR4). According to this, aFP plays a critical role in promoting the metastasis of HCC. Furthermore, aFP modulates the expression of PD-L1 and B7-H4, resulting in the immune escape of HCC and consequently strengthening its ability to metastasize [20,21].

The notion that cancer epithelial cells are responsible for metastases tends to be abolished [21]. On these grounds, the target of this study—in respect to CTC detection—was to determine the tumor cells that switch or are prone to switching towards a mesenchymal phenotype rather than to detect tumor epithelial cells. This may also explain the lower number of advanced HCC patients that present CTCs in their periphery.

EPCAM expression is observed in epithelial cells and in cancer cells at early stages of the epithelial to mesenchymal transition (EMT) [20,21,22]. Vimentin is a mesenchymal marker, and sMVP is a marker of intermediate differentiation towards mesenchymal phenotype [10,11,12,13].

CTCs were detected in 51% of patients with advanced HCC, in 29% of patients with early HCC and in 7% of cirrhotic patients. These patients presented evidence of epithelial CTCs and of mesenchymal subtypes as was determined by both techniques. From our findings, we can conclude that despite the fact that CTCs are considered to be associated with advanced cancer, are predictors of prognosis [10,11,12,13,23,24] and are suitable for patient monitoring, they are not suitable as a diagnostic tool for defining cirrhotic patients prone to develop early HCC or for defining early HCC patients. For the markers examined, CTCs seem to be a very sensitive prognostic marker but with rather low specificity since they are also observed in some cirrhotic patients. Our results concur with several other studies which demonstrated the presence of CTCs in at least 12% of cirrhotic patients [19,23].

With respect to miRNA analysis, miR-200a and miR-200b repression have been proposed as early markers of HCC development [16,25]. It has been demonstrated that miR-200b was highly expressed in the plasma-derived exosome of ovarian cancer patients, promoting the proliferation and invasion of cancer cells. The proposed mechanism is the induction of macrophage M2 polarization by suppressing KLF6 expression [25]. Furthermore, miR-200b was significantly upregulated in patients with early HCC compared to cirrhotic patients, and miR-200b abnormal expression in serum was related to tumor occurrence and development [26].

ΜiR-200b inhibits the epithelial to mesenchymal transition through ZEB1 and ZEB2 inhibition, allowing the expression of E-cadherin and leading to EMT inhibition. It also reduces angiogenesis and inhibits the Notch 1 signaling pathway [27]. The Notch 1 mode of action is controversial since initial evidence suggested it as a tumor suppressor. On the other hand, there is growing evidence indicating that Notch 1 overexpression may also exert an oncogenic effect [28].

In this study we found that patients with either early or advanced HCC presented a significant upregulation in miR-200b levels compared to the cirrhotic patients. HCC patients with CTCs exhibited significant upregulation of miR-200b that was proportional to the elevation of EPCAM.

Taking into account the stability of miRNAs, a major question that arises is if the levels of miR-200b in the blood remain constantly high so they can be detected and if they remain high despite the absence of CTCs in the blood. Our results are rather controversial when compared to other studies regarding the use of the miRNAs as useful prognostic and diagnostic markers for HCC [24,25,26,27,28,29]. It seems that the majority of the mRNAs used in this study, with the exception of miR-200b, apart from being present in a number of HCC patients independently from the presence of CTCs were also expressed in a number of cirrhotic patients (Table 3). Therefore, based on our evidence, they presented low specificity to be used as diagnostic biomarkers. 

In most proteomics studies in the literature, the comparison of biomarkers is amongst cirrhotic, and generally, HCC patients at any stage rather than cirrhotic and early HCC patients [30,31]. Our evidence suggests that advanced HCC patients present significantly different proteome profiles when compared to early HCC patients. Therefore, we compared the proteome profiles of non-HCC cirrhotic patients to those of patients with early HCC.

**Figure 6:**
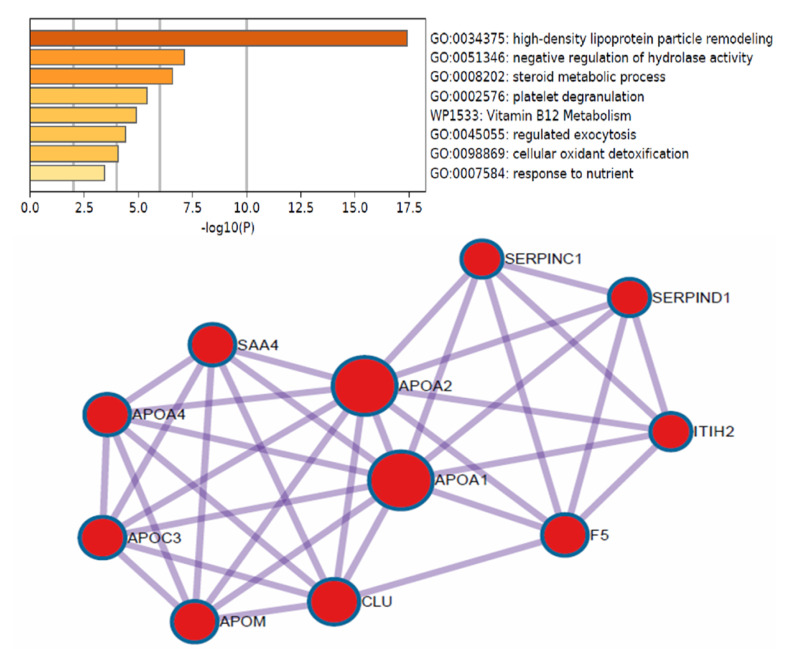
Enrichment analysis of the biological processes related to the proteins found from the proteomics analysis, where apolipoproteins seem to play a central role in hepatocellular carcinoma (Metascape.org, accessed on 21 August 2022).

Proteomics analysis of these individuals led to the identification of a total of 31 markers, which were upregulated in patients with early HCC compared to cirrhotic patients. These markers were selected in respect to their significance in respect to upregulation in patients with early HCC when compared to cirrhotic patients and to their significant involvement in the five major cancer-related functions examined (growth, immune response, angiogenesis, proliferation and metastases) as found in our study. Examination of the protein profile of each cirrhotic and early HCC patient suggests that most of the markers, despite the initial significance in the comparison between groups, may not be suitable as diagnostic markers since they seem to lack specificity in many instances. Ideally, a marker should be capable of identifying early HCC from cirrhotic patients. In this study, APOC3 protein—which was found to have one of the highest significant differences in the comparison between groups—was upregulated in 88% of patients with early HCC and in 2/28 (7%) cirrhotic patients, whereas APOA2 was found to be expressed in 85% of early HCC patients and in 2/29 (7%) cirrhotic patients. It is worth mentioning that when we examined both markers, we covered 100% of the patients with early HCC. Apolipoproteins (APOs) have been increasingly reported for their relationships with tumors [31]. In this study, several apolipoproteins seem to be related to the development of early HCC (Figure 6). It has been previously suggested that APOA2 can be used as a biomarker in HCC and in prostate cancer [30,32]. Normally, APOA2 lipoprotein is mainly produced in the liver and is found in plasma as a monomer, homodimer, or heterodimer with apolipoprotein D. These are all involved to the catalysis of lipoproteins towards high-density lipoprotein (HDL) [31].

APOC3 is an inhibitor of lipoprotein and hepatic lipases, and it has been proposed to inhibit the liver’s absorption of triglyceride-rich particles. At the cellular level, APOC3 appears to promote the assembly and secretion of triglyceride-rich low-density lipoprotein (VLDL) particles from hepatocytes under lipid-rich conditions [33]. In cancer, APOC3 induces the NLRP3 inflammasome via caspase 8 and toll-like receptors 2 and 4 [33]. Cytosolic caspase 8 is a mediator of death receptor signaling. Caspase 8 depletion induces G2/M arrest, p53 stabilization and induction of p53-dependent intrinsic apoptosis in tumor cells [34]. From the above, it can be understood that APOC3 is directly involved in cancer progression, whereas APOA2 is a newly recognized biomarker in HCC [30,31,32]. 

In our quest towards the determination of novel biomarkers that will define HCC at very early stages, our evidence suggests that a combined tool of three markers (miR-200b, APOA2 and APOC3) could diagnose HCC at very early stages accurately and with high sensitivity. Each of these markers separately presents greater sensitivity and specificity than aFP, the traditionally used marker for HCC [35]. The detection of elevated serum aFP levels in only 21% of early HCC observed in this study supports the above results. 

We acknowledge that this study has limitations, which are mainly the small sample size and the lack of a validation cohort. However, we believe that these preliminary data are significant and prompt further investigation to elucidate the role of the proposed molecules as a useful diagnostic tool for patients with cirrhosis prone to developing HCC.

## 5. Conclusions

Our evidence suggests that patients with early HCC present with a distinct proteome profile when compared to cirrhotic patients. The combined upregulation of APOA2 and APOC3 can be used as an accurate and sensitive diagnostic tool for the diagnosis of early HCC. Moreover, based on our findings, the increased levels of miR-200b in serum could also contribute to the identification of early HCC development. Finally, CTCs, although significantly increased in patients with advanced HCC in our study, showed low specificity in the distinction between HCC and non-HCC patients. Further confirmation of the above results in larger studies may highlight the utility of combined proteomics and molecular biomarkers in the timely diagnosis of early HCC and in individualized cancer management.

## Figures and Tables

**Table 1:** Patient Characteristics.

	HCC Patients (*n* = 89)	Cirrhotic Patients (*n* = 28)
Median age, years (range)	65 ± 11 years	60 ± 7 years
Male sex, *n* (%)	67, (75%)	20, (71%)
**Liver Disease, *n* (%)**		
Viral hepatitis	32, (36%)	9, (32%)
Non-viral hepatitis	57, (64%)	19, (68%)
**HCC stage, *n* (%)**		
BCLC A	33, (37%)	n/a
BCLC C	56, (63%)	
**Microvascular invasion**		
(Available data in 55 patients)	23, (26%)	n/a
**Serum aFP values >20 ng/ml**		
Early HCC	7/33 (21%)	-
Advanced HCC	42/56 (75%)	-
Cirrhotic	-	0/28 (0%)

HCC, hepatocelllar carcinoma; BCLC, Barcelona Clinic liver cancer staging system; AFP, alpha fetoprotein; n/a, not available.

**Table 2:** Synopsis of the results indicating the presence of CTCs in each group.

	EPCAM	Vimentin	AFP	sMVP
**Advanced HCC** (53 pts)	27/53	10/53	12/53	6/53
**Early HCC** (17 pts)	5/17	3/17	1/17	1/17
**Cirrhosis** (28 pts)	2/28	1/28	1/28	0/28

Pts: patients; aFP: alpha fetoprotein; EPCAM: epithelial cellular adhesion molecule; sMVP: surface major vault protein.

**Table 3:** miRNA upregulation in comparison with CTCs.

(i)				
	**EPCAM**	**Vimentin**	**AFP**	**sMVP**
**Cirrhotic**				
miR-200b	* 2/2	* 1/1	* 1/1	0/2
miR-122	* 2/2	* 1/1	0/1	0/2
miR-221	0/2	0/1	0/1	0/2
miR-222	0/2	0/1	0/1	0/2
miR-200a	0/2	0/1	0/1	0/2
**Early HCC**				
miR-200b	* 4/5	* 3/3	* 1/1	0/1
miR-122	* 3/5	* 1/3	0/1	0/1
miR-221	* 2/5	0/3	0/1	0/1
miR-222	* 3/5	* 1/3	0/1	0/1
miR-200a	0/5	0/3	0/1	0/1
**Advanced HCC**				
miR-200b	* 7/27	* 7/10	* 3/12	* 2/6
miR-122	* 11/27	* 9/10	* 7/12	* 4/6
miR-221	* 5/27	* 5/10	* 3/12	* 2/6
miR-222	* 12/27	* 8/10	* 10/12	* 4/6
miR-200a	* 3/27	* 3/10	* 3/12	0/6
(ii)				
**miR-200b**		Cirrhotic	Early HCC	Advanced HCC
CTCs		2	4	7
No CTCs		1	2	3
**miR-122**		Cirrhotic	Early HCC	Advanced HCC
CTCs		2	3	11
No CTCs		7	7	6
**miR-221**		Cirrhotic	Early HCC	Advanced HCC
CTCs		0	2	5
No CTCs		13	8	9
**miR-222**		Cirrhotic	Early HCC	Advanced HCC
CTCs		0	3	12
No CTCs		13	7	8
**miR-200a**		Cirrhotic	Early HCC	Advanced HCC
CTCs		0	0	3
No CTCs		7	4	6

* The patients that presented miRNA upregulation also presented EPCAM, vimentin, AFP and sMVP upregulation. (i) Pathological expression of different miRNAs in patients that presented CTCs of various subtypes (EPCAM, vimentin, sMVP). (ii) Correlation of CTCs with the pathological expression of miRNAs.

## Data Availability

The mass spectrometry proteomics data have been deposited to the ProteomeXchange Consortium via the PRIDE [1] partner repository with the dataset identifier PXD033306.

## Supplementary Materials

The following supporting information can be downloaded at: https://www.mdpi.com/article/10.3390/cimb44100322/s1. Table S1: Specific gene and primer sequence for the gene expression analysis. Figure S1: Determination of the significance of EPCAM, Vimentin and miR200b as biological markers for the determination of early HCC, Considering the area under the curve only miR200b seems to be a suitable molecule as a biomarker for early HCC diagnosis.

## List of Abbreviations

Hepatocellular carcinoma	(HCC)
microRNAs	(miRNAs)
Circulating tumor cells	(CTCs)
Epithelial cell adhesion molecule	(EPCAM)
Alpha fetoprotein	(aFP)
Surface major vault protein	(sMVP)
Chronic hepatitis B virus	(HBV)
Hepatitis C virus	(HCV)
Non-alcoholic steatohepatitis	(NASH)
Erythrocyte lysis buffer	(ELB)
Quantitative real-time polymerase chain reaction	(qRT-PCR)
Immunofluorescence staining	(IF)
4′,6-diamidino-2-phenylindole	(DAPI)
Liquid chromatography mass spectrometry	(LC MS)
Gas phase fractionation	(GFP)
Computed tomography/magnetic resonance imaging	(CT/MRI)
Matrix metalloproteinase 2/9	(MMP2/9)
C-X-C motif chemokine receptor 4	(CXCR4)
Apolipoprotein	(APO)
High-density lipoprotein	(HDL)
Triglyceride-rich low-density lipoprotein	(VLDL)
