# Supplementary material for: Cellular, Molecular and Proteomic Characteristics of Early Hepatocellular Carcinoma
Source: Curr Issues Mol Biol. 2022 Oct 10;44(10):4714–34. doi: 10.3390/cimb44100322 (PMC9600540; doi:10.3390/cimb44100322)
Supplement: Supplementary file 1 [file cimb-44-00322-s001.zip › cimb-1902648-supplementary.pdf]

Figure S1:Determination of the significance of EPCAM, Vimentin and miR200b as biological markers for the determination of early HCC, Considering the area under the curve only miR200b seems to be a suitable molecule as a biomarker for early HCC diagnosis.

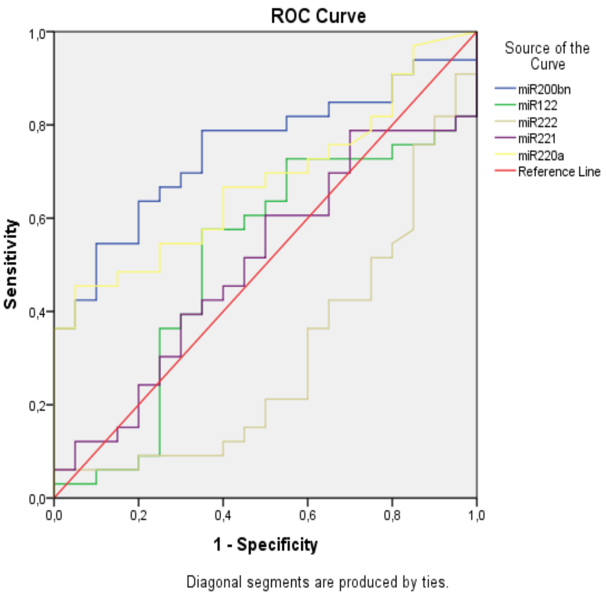

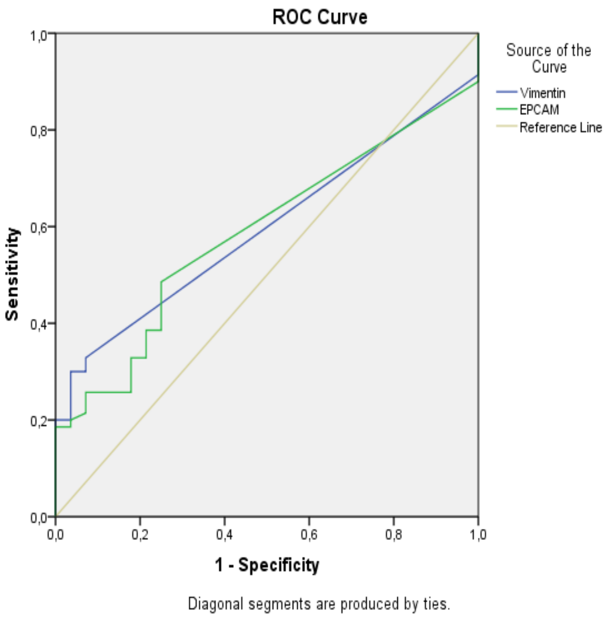

Area Under the Curve

Test Result Variable(s)	Area	Std. Error[a]	Asymptotic Sig.[b]	Asymptotic 95% Confidence Interval	
				Lower Bound	Upper Bound
Vimentin	,595	,057	,144	,484	,706
EPCAM	,587	,058	,181	,472	,701
miR200b	,747	,067	,003	,616	,818
miR122	,509	,085	,912	,343	,675
miR222	,304	,076	,017	,154,	,453
miR221	,503	,081	,971	,344	,662
miR200a	,670	,072	,030	,547	,821

Biomarker	***Cut-off***	***sensitivity***	**1-specificity**
**EPCAM**	2,16	40%	25%
**Vimentin**	4,8	30%	36%
**miR200b**	0,094	70%	30%

The test result variable(s): Vimentin, EPCAM has at least one tiebetween the positive actual state group and the negative actual state group. Statistics may be biased.

a. Under the nonparametric assumption

b. Null hypothesis: true area = 0.5

Supplementary Table S1: Specific genes and primers sequence for the gene expression analysis.

Full Name	Primer	Accession Number	Forward primer	Reverse primer
Homo sapiens miR-122-5p	**miR122**	MI0000442	TGGAGTGTGACAATGGTGTTTG	
Homo sapiens miR-221-3p	**miR221**	MI0000298	AGCTACATTGTCTGCTGGGTTTC	
Homo sapiens miR-222-3p	**miR222**	MI0000299	AGCTACATCTGGCTACTGGGT	
Homo sapiens miR-200a-3p	**miR200a**	MI0000737	TAACACTGTCTGGTAACGATGT	
Homo sapiens miR-200b-3p	**miR200b**	MI0000342	TAATACTGCCTGGTAATGATGA	
Homo sapiens Major vault protein (MVP)	**MVP**	NM_017458.3	GAGGGACCTGGCACGTACA	TACGCCCCTACTGTGGTGAC
Homo sapiens epithelial cell adhesion molecule (EPCAM),	**EPCAM**	NM_002354.3	GCCAGTGTACTTCAGTTGGTGC	CCCTTCAGGTTTTGCTCTTCTCC
Homo sapiens Vimentin	**VIM**	NM_003380	CTAACCAACGACAAAGCCC	CCTCTCTCTGAAGCATCTCC
Homo sapiens Alpha fetoprotein (AFP)	**AFP**	NM_001134.3	CCGAACTTTCCAAGCCATAAC	GACAATCCAGCACATCTCC
Homo sapiens glyceraldehyde-3-phosphate dehydrogenase (GAPDH)	**GAPDH**	NM_002046	CAACGGATTTGGTCGTATT	GATGGCAACAATATCCACTT
